# Exploring the Bacterial Microbiota of Seeds

**DOI:** 10.1111/1751-7915.70230

**Published:** 2025-09-10

**Authors:** Angel J. Matilla

**Affiliations:** ^1^ Departamento de Biología Funcional Universidad de Santiago de Compostela Santiago de Compostela Spain

## Abstract

The seed microbiota, a still underexplored component of plant–microbe interactions, plays a pivotal role in plant development and holds significant promise for advancing sustainable agriculture. By influencing essential processes such as germination, stress tolerance, nutrient acquisition and defence, seed‐associated microbes offer unique advantages beyond those of soil‐ or rhizosphere‐associated microbiomes. Notably, they are transmitted both vertically and horizontally; however, fundamental questions remain regarding their origin, ecological dynamics and functional roles across environments. This article explores the diversification of seed microbiota as a consequence of crop domestication, emerging insights into functional microbial genes and key challenges that must be addressed to fully unlock its potential.

## Graphical abstract

The presence of a functional bacterial microbiota in seeds is well established. However, the mechanisms underlying microbial entry into the propagule and its intracellular compartments remain largely unresolved.

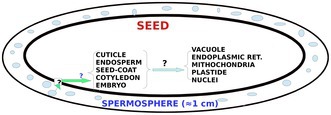

## 1 An Updated Background on Seed Microbiome Diversity

It is widely accepted that microbial communities are involved throughout the plant's life cycle and colonise all its organs (Trivedi et al. 2020; Joubert et al. 2024). That is, during their life cycle, higher plants interact with various microbial communities, many of which are inherited by the next generation via seed transmission (Nelson 2018). The term *microbiota* or *phytomicrobiome* refers to microorganisms residing inside (endophytic) and on the surface (epiphytic) of plants (e.g., root, stem, leaf, flower and seeds). This collective community of microbes influences plant growth, organ development, stress resilience and overall health (Hassani et al. 2018; Nelson 2018; Simonin, Barret, et al. 2022; Anand et al. 2023). Most studies focus on the rhizosphere microbiome, which mediates root–soil interactions essential for nutrient uptake and plant health. Among plant organs, seeds have historically been the least studied in terms of their associated microbiota, despite their critical role as ecological niches, sources of microbial diversity and key vectors for vertical transmission of beneficial microbes. However, the seed microbiome is a significant reservoir of beneficial microorganisms that influence key processes such as seed dormancy, germination, growth promotion and other critical aspects of plant development (Table 1). Interestingly, Samreen et al. (2021) emphasised the significance of the seed microbiome in determining seed quality. On the other hand, recent findings indicate a strong link between seed quality and its endophytic content. Thus, seeds enriched with diverse endophytic communities tend to show higher germination rates, increased vigour and better stress tolerance (Sahu et al. 2023).

**TABLE 1:** Examples of crop–seed microbiomes.

Crop species	Key bacterial genera found in seeds	Important functions
Wheat	*Bacillus*, *Pseudomonas*, *Paenibacillus*	Growth promotion, antifungal effects
Maize	*Enterobacter, Azospirillum*	N_2_ fixation, hormone production
Rice	*Burkholderia*, *Rhizobium*, *Bacillus*	Stress tolerance, biocontrol
Soybean	*Bradyrhizobium*, *Streptomyces*	Symbiotic N_2_ fixation

Let us keep in mind that seeds are not sterile structures; instead, they harbour diverse microbial communities composed of bacteria, fungi and other microorganisms. In this context, the seed microbiome was first documented by Samish and Etinger‐Tulczynska (1963), who detected microorganisms within seeds during their analysis of tomato fruit. Compared to the microbiomes of other plant parts, only a small fraction of microbial taxa successfully colonise seeds (Tarquinio et al. 2021). Nevertheless, substantial progress in the study of seed‐associated (SdA) microbiota emerged in the early decades of the 21st century, coinciding with significant advances in our understanding of the seed life cycle (Firdous et al. 2019; Choi et al. 2021; Kim et al. 2023; Matilla et al. 2023; Kumar et al. 2024, see Table 2; Matilla 2025 for a comprehensive review). Several studies have revealed that seeds harbour microbial communities composed of taxa from diverse phyla, particularly Proteobacteria, Actinobacteria and Firmicutes (Guo et al. 2021; Simonin, Chesneau, et al. 2022; War et al. 2023). Interestingly, bacteria belonging to the genera *Bifidobacterium*, *Faecalibacterium*, *Lactobacillus*, *Clostridium* and *Streptococcus*, commonly known as animal gut residents, have also been identified as abundant members of the epiphytic seed microbiome (Grond et al. 2018; Morales‐Moreira et al. 2021). That is, seeds harbour diverse microbial communities, as demonstrated by techniques such as fluorescence microscopy and in situ hybridisation (Sharma et al. 2023; Kumar et al. 2024). However, a large proportion of microbial taxa cannot be cultured using conventional laboratory methods. To overcome this limitation, metagenomics, Next‐Generation Sequencing (NGS) and 16S rRNA gene sequencing have become indispensable for exploring the taxonomic and functional diversity of SdA microbiota.

**TABLE 2:** Distribution of functional genes in natural seed microbiomes, artificially inoculated seeds and rhizosphere microbial communities surrounding seeds.

Action	Gene	Bacteria
Solubilisation of Pi^a^	*pqq, gcd*	*Pantoea*, *Bacillus*, *Flavobacterium*
Production of GAs, ET, CKs^b^	*acds*	*Bacillus, Pseudomonas, Enterobacter*
Production of auxin^c^	*ipdC, iaaM, iaaH*	*Azospirillum*, *Enterobacter, Bacillus, Pseudomonas*
Lytic enzymes production^d^	*chiA*	*Trichoderma* spp., *Bacillus*, *Pseudomonas*

^a^ Metagenomic work on the maize plant‐associated seed microbiome (Henaut‐Jacobs et al. 2024); *pqq*: pyrroloquinoline quinone; *gcd*: glucose dehydrogenase.

^b^ In plant‐associated bacteria, with functional evidence both in the rhizosphere and inoculated seeds with synthetic microbial consortia or bioformulations (Nascimento et al. 2018, 2019); *acds*: 1‐aminocyclopropane‐1‐carboxylate deaminase.

^c^ In artificially inoculated seed, and the rhizosphere. No confirmation in natural seeds (Figueredo et al. 2023); *ipdc*: indole‐3‐pyruvate decarboxylase; *iaaM*: tryptophan monooxygenase; *iaaH*: indole‐3‐acetamide.

^d^ *chiA* is rarely detected in natural seed microbiomes. However, seed‐applied *Trichoderma*, *Bacillus* and *Pseudomonas* show chitinase activity linked to biocontrol and SR (Brotman et al. 2012); *chiA*: chitinase A.

Despite these advances, little is known about how these microbes are acquired or their specific effects on seed germination and plant growth. Seed microbial transmission can occur vertically, from the mother plant, or horizontally, from the surrounding environment (Frank et al. 2017; War et al. 2023). Transmission pathways include internal routes via reproductive tissues (e.g., seed structure), floral pathways and external surfaces (Romao et al. 2025). However, the mechanisms governing microbial transmission, their functional roles and their impact on plant development remain poorly understood (reviewed in Jha et al. 2025). Four recent studies from Simonin's group collectively underscore the crucial role of seed microbiota in plant health and development. A large‐scale meta‐analysis identified a core group (i.e., a stable fraction of microbial taxa) of microorganisms consistently shared across plant species, which appears to be essential for seedling establishment and plant health and productivity. Research has revealed that seed microbial diversity is elevated and similar to that found in rhizosphere soil. Analyses of bacterial diversity across seeds from various plant species revealed the presence of microbes from over 200 genera spanning 11 phyla, with Proteobacteria being the most dominant phylum, encompassing 125 genera (War et al. 2023; see phylogenetic tree in Figure 1a). The most abundant and prevalent taxa comprising the core seed microbiome belonged to the genera *Pseudomonas*, *Pantoea*, *Enterobacter*, *Alternaria*, *Cladosporium* and *Fusarium* (Guo et al. 2021; Simonin, Barret, et al. 2022).

In addition, several studies have demonstrated that certain microbes are consistently transmitted from parent plants to seedlings, establishing seeds as key vectors of microbial inheritance. Moreover, recent research has shown that inoculating seeds with synthetic bacterial communities, along with understanding the ecological processes governing seedling colonisation, can support the development of effective microbiome engineering strategies from the earliest stages of plant growth (Chesneau et al. 2022; Simonin, Barret, et al. 2022; Arnault et al. 2024; Joubert et al. 2024). Recently, Singh's group introduced biopriming as a biotechnological strategy to improve seed performance (Singh et al. 2023). This method involves treating seeds with beneficial microbes (e.g., *Bacillus subtilis* , *Pseudomonas fluorescens* , *Trichoderma* spp., *Paenibacillus* spp. and *Enterobacter* spp.) and applying controlled hydration to enhance germination, protect against soil‐borne pathogens and contaminants and improve both crop productivity and soil health. Overall, biopriming stands out as a key technique for driving sustainable agricultural development. Microbes used for priming can be transmitted vertically from one generation to the next, and in some cases, are conserved across successive generations (Kim et al. 2022). However, although biopriming shows great potential, its practical application is still limited, requiring further research, standardisation and broader adoption by farmers (Srivastava et al. 2024; Janah et al. 2025).

A recent review hypothesised that harnessing beneficial SdA microbes can enhance germination, support plant establishment and contribute to sustainable agriculture (Magaña‐Ugarte et al. 2024; Jha et al. 2025). Moreover, seeds play a crucial role in microbial dispersal (Frank et al. 2017). In other words, SdA microbial communities are dynamic and remain metabolically or functionally active throughout subsequent stages of plant ontogeny (Nelson 2018). These microbial interactions are particularly critical during the early and vulnerable stages of plant development, playing a key role in seedling establishment and overall growth (Truyens, Jambon, et al. 2015; Yao et al. 2024). Similarly, seed microbiome diversity varies depending on plant genotype, season and developmental stage (Truyens, Jambon, et al. 2015; Jha et al. 2025). Interestingly, the seed microbiota composition of *Brassica napus* is cultivar‐dependent and primarily inherited through the patternal line (Wassermann et al. 2022). The findings of this research open new opportunities and provide valuable insights for integrating microbial traits into breeding programmes aimed at enhancing seed quality, crop productivity and plant resilience, as well as for designing targeted bacterial treatments.

The seed is the reproductive propagule used by Spermatophytes (seed plants) to transfer genetic material from one generation to the next (Sripathy and Groot 2023). In agricultural systems, seeds primarily serve to initiate new crop cycles. However, in natural ecosystems, seeds play broader ecological roles, facilitating not only reproduction but also dispersal, adaptation and establishment in diverse environments (Beckman and Sullivan 2023). This essential structure, which plays a fundamental role in the life cycle of higher plants, is composed of multiple functional parts, including the embryo, endosperm, cuticle and seed coat, that interact dynamically to regulate microbial invasion, seed maturation and dormancy, germination and seedling establishment (Panjak et al. 2024; Sajeev et al. 2024; Hyvärinen et al. 2025; Matilla 2025). Consequently, there is growing interest in understanding the role of SdA, particularly those associated with seeds, due to their critical importance in the plant life cycle and maturation process. Interestingly, a positive correlation exists between seed quality and their endophytic content (Sun et al. 2023).

## 2 The Plant Domestication Process and Seed Microbiome

Domestication involves the deliberate selection and breeding of wild plant species to develop traits beneficial for agricultural production, such as higher yields, improved tolerance to environmental stresses and easier harvesting. Modern wheat domestication and breeding have reduced the host plant's control over its rhizosphere microbiome, resulting in microbial communities that are more diverse but less distinct from the surrounding soil. This shift is accompanied by a decline in essential microbial functions related to plant growth and protection (Zhao et al. 2025).

During domestication, seed dormancy has been reduced or eliminated to promote quicker and more uniform germination; however, this has increased the risk of pre‐harvest sprouting (Matilla 2024). That is, domestication and selective breeding of cultivated species have substantially altered seed traits and their associated microbiomes, with significant implications for germination, plant health and environmental resilience (Escudero‐Martínez and Bulgarelli 2023; Wagner et al. 2023; Soldan et al. 2024).

Because seeds transmit beneficial microbes, they provide an excellent model system for investigating the effects of domestication on microbial community structure and function (Smith et al. 2025). Nevertheless, few studies have specifically focused on the impact of domestication on seed microbiomes. Moreover, cereal domestication has increased bacterial diversity in seeds while reducing the complexity of microbial interactions, underscoring its profound influence on microbiome structure and presenting new opportunities for sustainable agricultural applications (Pérez‐Jaramillo et al. 2019; Abdullaeva et al. 2021; Bziuk et al. 2021; Zhao et al. 2025).

That is, wild species exhibited more structured and mature microbial interaction networks compared to domesticated ones. In other words, wild cereals display more complex and balanced microbial networks, featuring both bacterial and fungal hubs. In contrast, domesticated cereals (e.g., *Triticum* and *Hordeum*) have less connected, bacteria‐dominated networks (Abdullaeva et al. 2021). Recent studies support the idea of interkingdom microbial exchange during plant domestication rather than the possibility of contamination.

Thus, Kuzniar et al. (2020) analysed the microbiota of eight wheat seed cultivars across different seed compartments (i.e., embryo, endosperm and seed coat) and found the genus *Cutibacterium* present in all parts examined. This finding aligns with recent studies highlighting the complex distribution of microbial communities within seed tissues (Zhao et al. 2025).

In summary, domestication (i) drives shifts in microbial diversity, community structure and interactions within cereal seeds, highlighting a tight evolutionary link between plants and their associated microbiota; that is, domestication consistently and predictably influences the seed‐associated microbial community (Wagner et al. 2023); and (ii) not only shapes the physical traits of plants but also consistently affects their seed microbiota. These patterns could be leveraged to enhance crop resilience, productivity and sustainability. It is now known that the SdA microbiota critically shapes germination, seedling vigour and early plant fitness (Ren et al. 2023; Romao et al. 2025).

## 3 Seed‐Associated Microbiota: Functional Genes and Agronomic Relevance

The term *microbiota* has been used to describe diverse microbial communities across different hosts and environments (Boon et al. 2014). These authors emphasised the functional importance of such interactions by suggesting that the microbiome should be defined based on the collective genes present, rather than solely on taxonomy. It is now well established that a significant number of these genes (see Table 2) enhance seed quality by promoting germination and seedling vigour. Collectively, they enable seed‐associated microbes to function as biological growth promoters, contributing to improved physiological quality and overall seed performance.

### 3.1 Functional Genes and Their Role in Seed Quality

Microbial functional genes in and around seeds are essential for early plant development, driving growth, enhancing stress resilience and protecting against pathogens. However, this reproductive propagule remains one of the least characterised plant structures in terms of its microbiota, despite its central role in transmitting beneficial microorganisms across generations and initiating new plant life cycles. This knowledge gap is particularly evident in crop species, where both seed‐associated microbial communities and their genes interact to influence early development, stress tolerance and overall crop productivity (Table 1). Understanding seed‐associated microbial communities in major crops such as wheat, maize and rice is essential for advancing sustainable agriculture and improving plant health (Truyens, Weyens, et al. 2015; Simonin, Barret, et al. 2022; Simonin, Chesneau, et al. 2022). The limited attention it has received contrasts with the growing recognition of its ecological and agricultural significance as a microbial reservoir and transmission hub (Nelson 2018; Simonin, Chesneau, et al. 2022).

### 3.2 Epiphytic vs. Endophytic Microbial Communities in Seeds

Until recently, microorganisms on the seed surface were largely overlooked. However, Links et al. (2014) revealed that seeds of *Triticum* and *Brassica* host abundant and similar epiphytic bacterial communities, with up to 10^8^ genomes per gram, and that epiphytic fungi were dominated by pathogens like *Phoma*, *Alternaria* and *Fusarium*. In contrast, endophytic communities were distinct and dominated by Proteobacteria (≈88%) such as *Pantoea agglomerans* (see Michl et al. 2024 for *Thinopyrum intermedium* ). Unlike epiphytic bacteria, endophytic microorganisms form a robust and long‐lasting relationship with their host plants (Samreen et al. 2021).

### 3.3 Microbial Transmission and Assembly in the Spermosphere

The first plant microbiome is established during seed germination through vertical inheritance and horizontal acquisition from the surrounding environment (Shade et al. 2017). During this process, the spermosphere—a dynamic zone of microbial activity—forms around the seed as it imbibes water and releases nutrient‐rich exudates (Nelson 2018). SdA bacteria perform functions similar to those in the rhizosphere and can rapidly spread beneficial microbes through seeds (Smith and Lee 2020; Ai et al. 2023). The seed surface is often colonised via horizontal transfer from fruits, threshing residues, or soil (Jha et al. 2025).

In addition to their structural and physiological roles, seeds also harbour a diverse microbiome, consisting of bacteria, fungi and other microorganisms located either internally or externally. This SdA microbiome has been shown to influence seed health (Kumar et al. 2024), germination (Magaña‐Ugarte et al. 2024) and early plant development and is increasingly recognised as a key factor in plant fitness and resilience (Nelson 2018; Shade et al. 2017). These microbes are not merely passive inhabitants; they may be vertically transmitted and play essential roles in germination, seedling development and plant health.

Recent studies highlight the functional contributions of SdA microbiota in promoting plant growth, enhancing pathogen resistance and improving tolerance to abiotic stress (Pal et al. 2021; Mao et al. 2023; Kumar et al. 2024). Seeds acquire endophytes both from soil via the vascular system and from flowers via floral pathways (U'Ren and Zimmerman 2021; Chen et al. 2024). In maize, seed endophytes form a conserved and heritable microbiome that enhances plant growth and pathogen resistance (Pal et al. 2021; Wallace 2022). However, their effects can be context‐dependent, emphasising the need for careful selection for application in sustainable agriculture.

### 3.4 Ecological and Agricultural Implications

Notably, native SdA strains exhibit greater potential to improve drought tolerance in maize compared to non‐native strains, underscoring the importance of selecting locally adapted microbial consortia for bioinoculants in water‐limited environments (Gil et al. 2024). Furthermore, seeds of hyperaccumulator plants serve as reservoirs of endophytes capable of resisting heavy metals, supporting germination and enhancing stress tolerance in contaminated soils (Zhang et al. 2024).

The reviews by War et al. (2023) and Kumar et al. (2024) underscore the crucial ecological and agricultural roles of seed microbiomes, highlighting their impact on plant health, adaptation and sustainability. Garrido‐Sanz and Keel (2025) recently provided compelling evidence that wheat seed‐borne bacteria influence rhizosphere assembly by occupying key ecological niches and facilitating colonisation by other beneficial microbes. Similarly, Hu et al. (2025) concluded that seed‐borne endophytes play vital roles in plant development, stress tolerance and microbial inheritance. While many are beneficial, some may harbour latent pathogens, necessitating further study.

Seed endophytic bacteria have been widely employed in sustainable agriculture as biocontrol agents, biofertilizers and inducers of abiotic stress tolerance during the past few decades. Bacteria such as *Pantoea* sp., *Citrobacter* sp., *Bacillus* sp. and *Flavobacterium* sp. can enhance nutrient solubilisation (Jana et al. 2023). Seed endophytes can produce subtilomycin, which binds to flagella to influence the plant defence induced by the flagellin peptide. Alternatively, they can accumulate PR1 protein, which triggers the WRKY53 gene expression through the jasmonic acid and ethylene signalling pathway and activates MAPK signalling (Mengistu 2020).

In summary, understanding the composition, transmission and ecological functions of the seed microbiome offers promising opportunities for developing sustainable agricultural practices and microbiome‐based seed treatments. As highlighted by recent reviews (War et al. 2023), this knowledge is essential not only for improving crop resilience but also for enhancing ecosystem sustainability and promoting climate‐smart agriculture.

## 4 Challenges for the Future

- Future research should focus on understanding how bacterial communities assemble and are transmitted within seeds and on identifying their specific roles in germination, plant growth and stress resistance (e.g., drought). Key priorities include studying plant genotype–microbiome interactions, developing seed‐based microbial inoculants, preserving beneficial seed microbiota in germplasm banks and utilising locally adapted strains for climate‐resilient agriculture. These efforts aim to translate microbiome knowledge into sustainable agricultural innovations.
- To unlock the full potential of seed microbiota, future research must address how microbes are distributed, transmitted and function across different seed compartments—and how this knowledge can be applied to crop improvement. In other words, it is necessary to investigate how bacterial communities assemble within the seed during plant development.
- One important objective should be to investigate at the molecular level the effects of endophytic and ectophytic bacteria on key molecular processes involved in dormancy, after‐ripening and germination in both crop seeds and model species such as Arabidopsis and rice. Integrated‐omics approaches (metagenomics, transcriptomics and metabolomics) and genetic editing tools (CRISPR, microbial mutants) should be employed to advance our understanding.
- It remains unclear how seeds and/or their associated bacteria are able to coordinate and prioritise among the key physiological processes mentioned in (iii) at specific stages of the seed life cycle. Therefore, it is essential, among other aspects, to understand how plant genotype influences the composition and stability of the seed microbiome.

## Author Contributions

**Angel J. Matilla:** conceptualization, writing – original draft, writing – review and editing.

## Conflicts of Interest

The author declares no conflicts of interest.

## Data Availability

The data that support the findings of this study are available from the corresponding author upon reasonable request.
